# Data on cut-edge for spatial clustering based on proximity graphs

**DOI:** 10.1016/j.dib.2019.104899

**Published:** 2019-11-29

**Authors:** Alper Aksac, Tansel Ozyer, Reda Alhajj

**Affiliations:** a Department of Computer Science, University of Calgary, Calgary, AB, Canada; b TOBB University of Economics and Technology, Ankara, Turkey; c Department of Computer Engineering, Istanbul Medipol University, Istanbul, Turkey

**Keywords:** Spatial data mining, Clustering, Proximity graphs, Graph theory

## Abstract

Cluster analysis plays a significant role regarding automating such a knowledge discovery process in spatial data mining. A good clustering algorithm supports two essential conditions, namely high intra-cluster similarity and low inter-cluster similarity. Maximized intra-cluster/within-cluster similarity produces low distances between data points inside the same cluster. However, minimized inter-cluster/between-cluster similarity increases the distance between data points in different clusters by furthering them apart from each other. We previously presented a spatial clustering algorithm, abbreviated CutESC (Cut-Edge for Spatial Clustering) with a graph-based approach. The data presented in this article is related to and supportive to the research paper entitled “CutESC: Cutting edge spatial clustering technique based on proximity graphs” (Aksac et al., 2019) [1], where interpretation research data presented here is available. In this article, we share the parametric version of our algorithm named CutESC-P, the best parameter settings for the experiments, the additional analyses and some additional information related to the proposed algorithm (CutESC) in [1].

**Table:** Specifications Table

Subject	*Computer Science (General)*
Specific subject area	*Spatial Data Mining, Clustering, Proximity Graphs, Graph Theory*
Type of data	*Table* *Figure*
How data was acquired	*Clustering analysis*
Data format	raw and analyzed
Experimental factors	*A preprocessing step is used for heterogeneous features. manuscript. The features are standardized by subtracting the mean and scaling to unit variance; all features are centered around zero.*
Experimental features	*Several clustering algorithms used to cluster various synthetic and real-world datasets from UCI repository, as well as real data related to image segmentation problems.*
Data source location	*Institution: University of Calgary* *City/Town/Region: Calgary, AB* *Country: CANADA*
Data accessibility	*The raw data files are provided in the Mendeley Data,*https://doi.org/10.17632/hkkbnxf4yp.1 [2]*. All other data is with this article.*
Related research article	*Alper Aksac, Tansel Özyer, Reda Alhajj* *CutESC: Cutting edge spatial clustering technique based on proximity graphs* *Pattern Recognition*https://doi.org/10.1016/j.patcog.2019.06.014

**Table:** 

**Value of the Data** • The parametric version of our algorithm presented here may be useful for users to set two parameters to better adapt clustering solutions for particular problems. • This data file presents the best parameter settings used in the experiments, which are helpful for researchers to enhance reproducibility and/or reanalysis. • This data file will be helpful to understand the CutESC algorithm in detail by providing additional information and experiments. • This approach works without any prior information and preliminary parameter settings while automatically discovering clusters with non-uniform densities, arbitrary shapes, and outliers.

## 1 Data

**Fig. 1:**
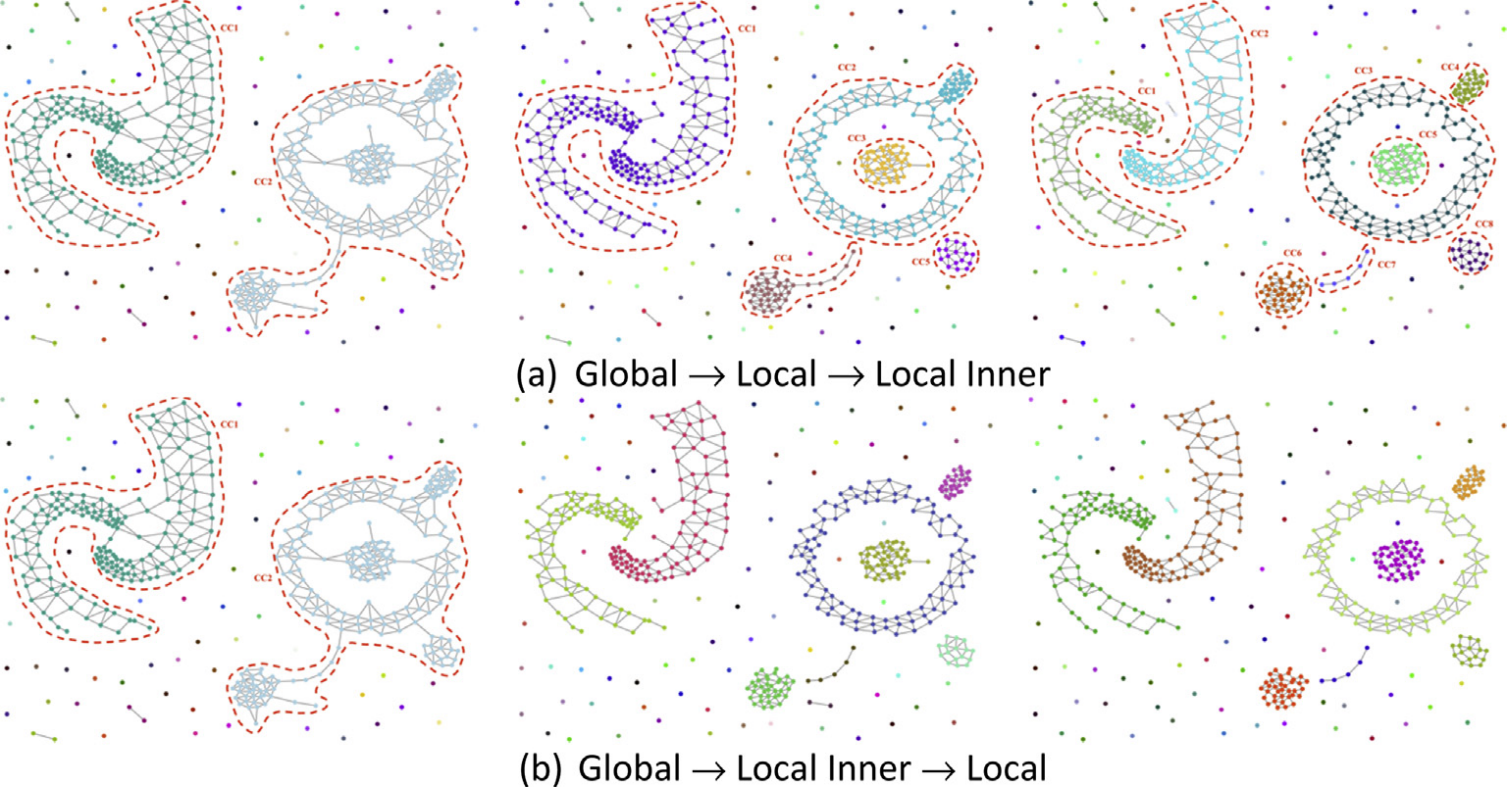
Second and third thresholding rules of the algorithm are applied in a flipped order. The algorithm mainly follows a top-down approach, where it first removed global (large scale effect) and later removed local edges (small scale effect), and global level → connected components (sub-groups) level → neighborhood level. The third rule provides more details to be considered using second order neighborhood, it is a pruning step for touching problems such as chain and necks. In the last stage of Fig. 1b, it can be seen that the touching problem (between green connected components (CC) and brown CC) could not be resolved.

**Fig. 2:**
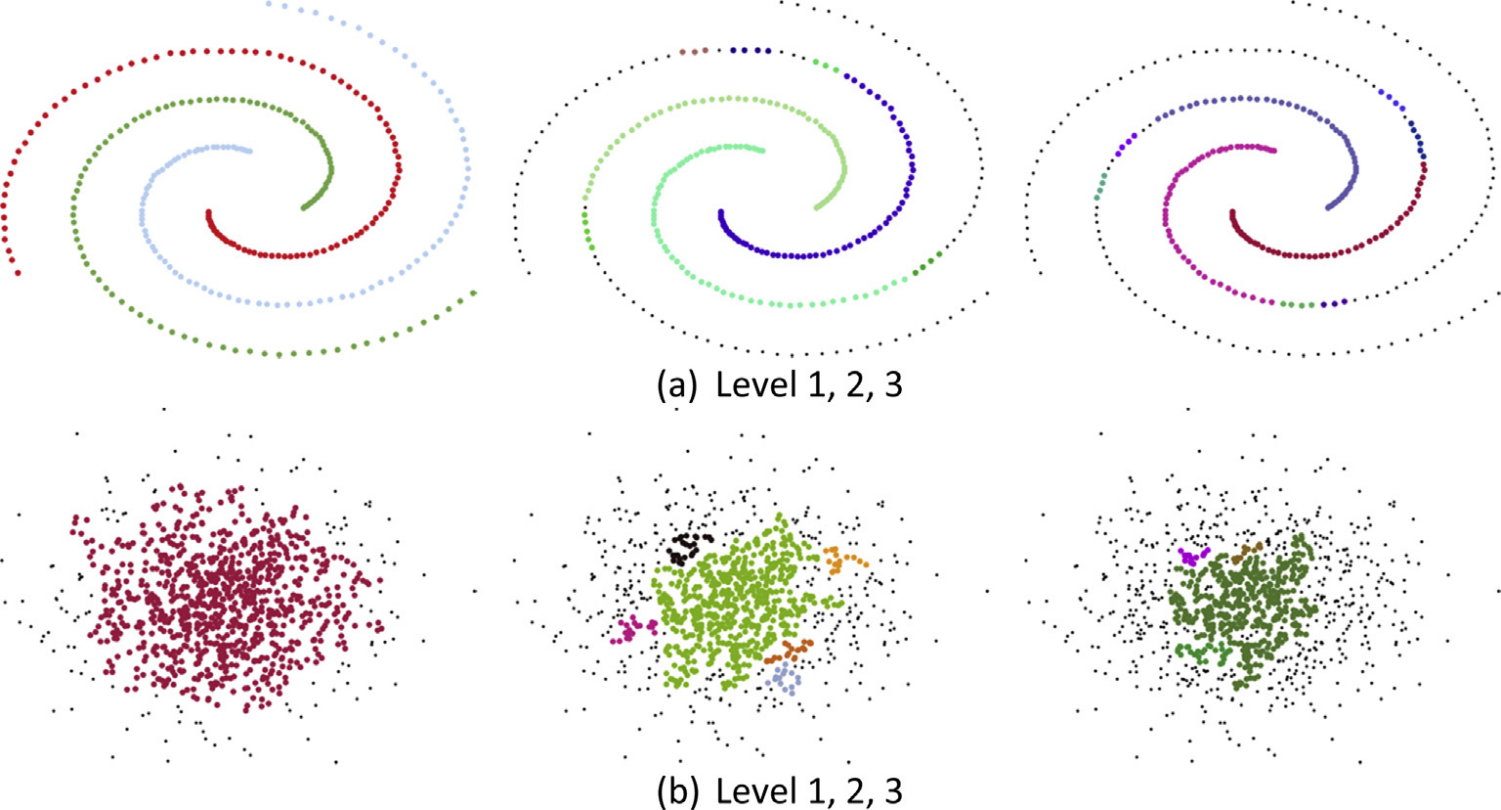
Our experiments with different cases show that one iteration is sufficient. It is also a trade-off between uniform (see Fig. 2a) and non-uniform (see Fig. 2b) scenarios. When the data become more chaotic, the useful information might be hidden in deeper levels and the algorithm needs to be run more than one iteration. We also provided this option to users for their special applications (see Algorithm 1 in Section 2.1).

**Fig. 3:**
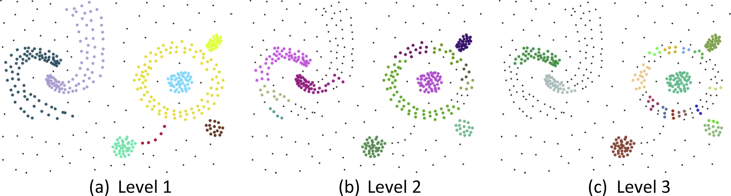
Running 3 iterations on the synthetic dataset [2] which is used to describe steps of the CutESC algorithm in the paper [1].

This article provides details about a novel algorithm (CutESC) for spatial clustering based on proximity graphs introduced in Ref. [1]. Moreover, the data in this article describes tables and figures in support of the article titled “CutESC: Cutting edge spatial clustering technique based on proximity graphs” [1]. CutESC performs clustering automatically for non-uniform densities, arbitrary shapes, and outliers without requiring any prior information and preliminary parameters. Besides, the parametric version of our algorithm (CutESC-P, see Algorithm 1 in 2.1) optionally allows interested users to tune the clustering process by setting two parameters for specific applications. In 2.1, CutESC-P refers to the parametric version of our algorithm. Some additional information related to the CutESC algorithm is provided in 2.2. The 3 thresholding procedures are presented so as to be in a hierarchy. Fig. 1 shows that second and third thresholding rules of the CutESC algorithm are applied in a flipped order. Fig. 2, Fig. 3 show that the CutESC algorithm obtains the optimal solution in the first iteration. The relation between levels is given at Table 1 where the number of clusters and Calinski-Harabasz score are shown for each level. We scanned through combinations of values for each algorithm. The best parameter settings for the experiments are given in 2.3. In the pre-processing step, features are standardized by subtracting the mean and scaling to unit variance. All features are centered around zero. We scanned through combinations of values for each algorithm to find the best parameter settings. Table 2 shows selected parameters for 3-spiral [5], Aggregation [6], Compound [7], D31 [8], Zelnik4 [9] datasets. Table 3 shows selected parameters for Chameleon [3] dataset. Table 4 shows selected parameters for UCI (Dermatology, Ionosphere, Heart-Statlog, Cardiac-Arrhythmia, Thyroid-Allbp) [4] datasets. Table 5 shows selected parameters for BSDS500 [10] dataset. Table 6 shows selected parameters for Histological [11] dataset. Other details on external clustering criteria are reported in Table 7, Table 8 of 2.4. The additional analysis for Real-World datasets based on external clustering criteria is included in 2.5. Table 9 includes the comparison for Real-World datasets based on external clustering criteria. Table 10 includes the number of instances that were attributed to each cluster as compared with the ground truth for Real-World datasets. The external clustering criteria of the image segmentation datasets is given in Table 11, Table 12 of 2.6.

**Table 1:** Iterative/Nested experiments for Fig. 2, Fig. 3, respectively. The high density and high dimensional datasets will increase the execution time of clustering algorithms as in our case. It is a trade-off between accuracy and speed. As in shown Fig. 2, Fig. 3, the CutESC algorithm obtains the optimal solution in the first iteration. However, meaningful or useful clusters in the chaotic data might be hidden in deeper levels. Moreover, while branching to sub-clusters, the *goodness* of the resulting clusters should not decrease. Many cluster validation indices have been published in the literature. The CutESC algorithm uses the Calinski-Harabasz score to evaluate the *goodness* (see Algorithm 1). While this score is increasing, the iteration will continue. Here, not only one index but also the combination of indices could be used. The Calinski-Harabasz score is in the range [0, +∞], a higher score indicates better clustering. It considers the quality of the distribution of the within-cluster and the between-cluster to define the score. As seen in the table, Calinski-Harabasz scores do not change when iterating in the first case (see Fig. 2a), but the number of clusters is increasing. In the second example, the score increases, but then it decreases. The second level has better *goodness* than other levels (see Fig. 2b). In the last example, the score is constantly decreasing thus the iteration will stop in the first step.

	Level 1	Level 2	Level 3
# of Clusters	3	8	9
Calinski-Harabasz	6	6	6
# of Clusters	1	6	4
Calinski-Harabasz	1	18	8
# of Clusters	8	13	19
Calinski-Harabasz	105	57	25

**Table 2:** Selected Parameters for 3-spiral [5], Aggregation [6], Compound [7], D31 [8], Zelnik4 [9] datasets.

Dataset	HDBSCAN	DBSCAN	OPTICS
3-spiral	minClusterSize = 2	eps = 0.1, minPoints = 4	eps = 0.1, minPoints = 3
Aggregation	minClusterSize = 12	eps = 0.05, minPoints = 3	eps = 0.082, minPoints = 3
Compound	minClusterSize = 3	eps = 0.05, minPoints = 3	eps = 0.1, minPoints = 8
D31	minClusterSize = 6	eps = 0.016, minPoints = 3	eps = 0.013, minPoints = 2
Zelnik4	minClusterSize = 6	eps = 0.075, minPoints = 7	eps = 0.015, minPoints = 3
Scanning Range	(2:1:20)	(0.01:0.001:0.1), (3:1:10)	(0.01:0.001:0.1), (3:1:10)

**Table 3:** Selected Parameters for Chameleon [3] dataset.

Dataset	CutESC-P	HDBSCAN	DBSCAN	OPTICS
t4.8k	α = 1, β = 0.8	minClusterSize = 9	eps = 0.015, minPoints = 6	eps = 0.013, minPoints = 1
t5.8k	α = 1, β = 0.7	minClusterSize = 6	eps = 0.013, minPoints = 10	eps = 0.013, minPoints = 9
t7.10k	α = 0.7, β = 1	minClusterSize = 12	eps = 0.014, minPoints = 7	eps = 0.02, minPoints = 3
t8.8k	α = 1, β = 1	minClusterSize = 11	eps = 0.013, minPoints = 3	eps = 0.013, minPoints = 2
Scanning Range	(0.1:0.1:1), (0.1:0.1:1)	(2:1:20)	(0.01:0.001:0.2), (3:1:10)	(0.01:0.001:0.2), (3:1:10)

**Table 4:** Selected Parameters for UCI [4] datasets.

Dataset	HDBSCAN	DBSCAN	OPTICS
Dermatology	minClusterSize = 5	eps = 0.5, minPoints = 5	eps = 0.9, minPoints = 10
Ionosphere	minClusterSize = 10	eps = 0.3, minPoints = 10	eps = 0.1, minPoints = 5
Heart-Statlog	minClusterSize = 10	eps = 0.5, minPoints = 9	eps = 0.5, minPoints = 8
Cardiac-Arrhythmia	minClusterSize = 5	eps = 0.3, minPoints = 5	eps = 0.5, minPoints = 8
Thyroid-Allbp	minClusterSize = 10	eps = 0.3, minPoints = 10	eps = 0.2, minPoints = 10
Scanning Range	(2:1:10)	(0.1:0.1:1), (3:1:10)	(0.1:0.1:1), (3:1:10)

**Table 5:** Selected Parameters for BSDS500 [10] dataset.

Image Name	HDBSCAN	DBSCAN	OPTICS
8068	minClusterSize = 5	eps = 0.1, minPoints = 3	eps = 0.1, minPoints = 3
42049	minClusterSize = 7	eps = 0.03, minPoints = 3	eps = 0.03, minPoints = 3
108073	minClusterSize = 7	eps = 0.2, minPoints = 3	eps = 0.2, minPoints = 4
260058	minClusterSize = 4	eps = 0.2, minPoints = 3	eps = 0.2, minPoints = 4
300091	minClusterSize = 9	eps = 0.2, minPoints = 3	eps = 0.2, minPoints = 3
Scanning Range	(2:1:20)	(0.01:0.01:0.2), (3:1:10)	(0.01:0.01:0.2), (3:1:10)

**Table 6:** Selected Parameters for Histological [11] dataset.

Image Name	HDBSCAN	DBSCAN	OPTICS
ih2ycmuhwrgalo	minClusterSize = 16	eps = 0.1, minPoints = 3	eps = 0.15, minPoints = 3
pbphl1xujdvyx	minClusterSize = 13	eps = 0.3, minPoints = 3	eps = 0.25, minPoints = 3
ebvubdfxocisgny	minClusterSize = 13	eps = 0.5, minPoints = 3	eps = 0.25, minPoints = 3
0anzqyibfuc	minClusterSize = 8	eps = 0.65, minPoints = 3	eps = 0.65, minPoints = 2
4nkj5wqcqj	minClusterSize = 10	eps = 0.35, minPoints = 3	eps = 0.3, minPoints = 6
Scanning Range	(2:1:20)	(0.1:0.05:1), (3:1:10)	(0.1:0.05:1), (3:1:10)

**Table 7:** Comparison for 3-spiral, Aggregation, Compound, D31, Zelnik4 based on external clustering criteria.

Algorithm	3-spiral			Aggregation			Compound			D31			Zelnik4		
	F-M	ARI	AMI	F-M	ARI	AMI	F-M	ARI	AMI	F-M	ARI	AMI	F-M	ARI	AMI

CutESC	1	1	1	0.859	0.802	0.798	0.976	0.968	0.937	0.620	0.571	0.809	1	1	1
HDBSCAN	1	1	1	0.878	0.839	0.868	0.882	0.833	0.822	0.598	0.569	0.819	0.923	0.903	0.899
AUTOCLUST	0.610	0.442	0.476	0.865	0.809	0.799	0.946	0.927	0.905	0.665	0.628	0.813	0.872	0.836	0.649
GDD	1	1	1	0.865	0.809	0.799	0.959	0.944	0.907	0.294	0.109	0.338	0.992	0.990	0.984
DBSCAN	1	1	1	0.865	0.809	0.799	0.961	0.949	0.885	0.652	0.624	0.807	0.935	0.919	0.916
MeanShift	0.330	−0.005	−0.005	0.888	0.847	0.818	0.851	0.778	0.742	0.587	0.525	0.725	0.870	0.833	0.618
OPTICS	1	1	1	0.885	0.852	0.809	0.836	0.757	0.697	0.600	0.531	0.747	1	1	1

**Table 8:** Comparison for Chameleon datasets based on external clustering criteria.

Algorithm		t4.8k			t5.8k			t7.10k			t8.8k		
		F-M	ARI	AMI	F-M	ARI	AMI	F-M	ARI	AMI	F-M	ARI	AMI
CutESC		0.916	0.897	0.875	0.940	0.930	0.912	0.890	0.841	0.836	0.978	0.974	0.940
CutESC-P		0.968	0.961	0.935	0.956	0.948	0.924	0.958	0.949	0.936	0.978	0.974	0.940
HDBSCAN		0.958	0.950	0.908	0.926	0.913	0.876	0.953	0.944	0.933	0.937	0.924	0.901
AUTOCLUST		0.939	0.926	0.759	0.909	0.893	0.720	0.890	0.868	0.759	0.797	0.746	0.687
GDD		0.407	0.007	0.021	0.369	0.011	0.063	0.405	0.006	0.988	0.401	0.009	0.022
DBSCAN		0.955	0.946	0.889	0.651	0.595	0.657	0.982	0.978	0.958	0.959	0.950	0.865
MeanShift		0.604	0.512	0.550	0.814	0.777	0.788	0.534	0.440	0.575	0.538	0.402	0.438
OPTICS		0.952	0.943	0.832	0.650	0.594	0.657	0.963	0.955	0.831	0.959	0.950	0.868

**Table 9:** Comparison for Real-World datasets based on external clustering criteria. At the bottom of table, the number of groups detected after the proposed algorithm (CutESC) of each one of the 3 clustering criteria which are global edges, local edges and local inner edges, respectively.

Algorithm	Dermatology			Ionosphere			Heart-Statlog			Cardiac-Arrhythmia			Thyroid-Allbp		
	Jaccard	Precision	Recall	Jaccard	Precision	Recall	Jaccard	Precision	Recall	Jaccard	Precision	Recall	Jaccard	Precision	Recall

CutESC	0.555	0.585	0.915	0.570	0.612	0.892	0.495	0.505	0.959	0.356	0.360	0.967	0.335	0.399	0.675
HDBSCAN	0.417	0.511	0.693	0.379	0.577	0.526	0.384	0.537	0.575	0.323	0.323	1	0.061	0.485	0.066
DBSCAN	0.199	0.199	1	0.496	0.529	0.887	0.384	0.504	0.617	0.323	0.323	1	0.173	0.494	0.211
MeanShift	0.199	0.199	1	0.538	0.538	1	0.494	0.508	0.949	0.323	0.323	1	0.319	0.389	0.637
OPTICS	0.269	0.279	0.888	0.538	0.538	1	0.403	0.503	0.671	0.323	0.323	1	0.265	0.452	0.390
AUTOCLUST	–	–	–	–	–	–	–	–	–	–	–	–	–	–	–
GDD	–	–	–	–	–	–	–	–	–	–	–	–	–	–	–
CutESC	Step 1	Step 2	Step 3	Step 1	Step 2	Step 3	Step 1	Step 2	Step 3	Step 1	Step 2	Step 3	Step 1	Step 2	Step 3
# of groups	4	4	4	2	2	2	2	2	2	2	2	2	4	4	4

**Table 10:** The number of instances that were attributed to each cluster as compared with the ground truth. In this table, rows represent the true class while columns are the predicted class. The values are reported using the contingency matrix which is used in statistics to define association between two partitions. In a clustering problem, true label names and predicted ones do not need to be the same, the assumptions are unclear. The number of clusters might not even be the same as true classes. According to this table, Cardiac-Arrhythmia dataset has 13 true classes however it is reported 16 in the UCI repository. The reason is that 3 classes (1. Degree AtrioVentricular block, 2. Degree AV block, 3. Degree AV block) actually include 0 instances in the dataset.

True Class	Dermatology				Ionosphere		Heart-Statlog		Cardiac-Arrhythmia		Thyroid-Allbp			
	1	2	3	4	1	2	1	2	1	2	1	2	3	4

1	6	0	106	0	43	83	2	148	2	243	183	1228	154	67
2	2	59	0	0	0	225	4	116	1	24	25	65	1	0
3	4	0	0	68	–	–	–	–	0	3	8	265	1	1
4	0	49	0	0	–	–	–	–	0	2	1	29	1	0
5	2	50	0	0	–	–	–	–	8	1	38	718	3	12
6	20	0	0	0	–	–	–	–	5	45	–	–	–	–
7	–	–	–	–	–	–	–	–	0	4	–	–	–	–
8	–	–	–	–	–	–	–	–	0	5	–	–	–	–
9	–	–	–	–	–	–	–	–	2	20	–	–	–	–
10	–	–	–	–	–	–	–	–	6	38	–	–	–	–
11	–	–	–	–	–	–	–	–	5	10	–	–	–	–
12	–	–	–	–	–	–	–	–	0	15	–	–	–	–
13	–	–	–	–	–	–	–	–	3	10	–	–	–	–

**Table 11:** Comparison for 5 selected images from BSDS500 dataset based on external clustering criteria.

Algorithm	8068					42049					108073					260058					300091				
	Dice	Precision	Recall	ARI	AMI	Dice	Precision	Recall	ARI	AMI	Dice	Precision	Recall	ARI	AMI	Dice	Precision	Recall	ARI	AMI	Dice	Precision	Recall	ARI	AMI

CutESC	0.933	0.941	0.924	0.886	0.685	0.926	0.953	0.901	0.904	0.743	0.855	0.783	0.941	0.551	0.366	0.807	0.717	0.923	0.686	0.568	0.907	0.997	0.833	0.756	0.490
HDBSCAN	0.846	0.815	0.880	0.730	0.550	0.532	0.407	0.768	0.316	0.283	0.835	0.729	0.976	0.430	0.267	0.783	0.653	0.976	0.631	0.420	0.681	0.928	0.538	0.362	0.294
AUTOCLUST	0.735	0.612	0.919	0.475	0.416	0.474	0.318	0.934	0.177	0.222	0.836	0.781	0.899	0.511	0.375	0.854	0.784	0.937	0.767	0.613	0.905	0.980	0.840	0.743	0.534
GDD	0.853	0.801	0.912	0.737	0.592	0.378	0.290	0.546	0.091	0.142	0.834	0.797	0.876	0.528	0.284	0.769	0.667	0.909	0.618	0.464	0.750	0.883	0.652	0.406	0.354
DBSCAN	0.848	0.815	0.883	0.733	0.566	0.505	0.385	0.733	0.274	0.253	0.861	0.795	0.940	0.576	0.341	0.806	0.703	0.945	0.680	0.471	0.886	0.977	0.810	0.701	0.484
MeanShift	0.840	0.818	0.863	0.723	0.522	0.525	0.389	0.807	0.294	0.304	0.839	0.744	0.963	0.465	0.284	0.708	0.718	0.697	0.558	0.456	0.623	0.903	0.475	0.288	0.209
OPTICS	0.845	0.813	0.880	0.729	0.562	0.494	0.371	0.741	0.253	0.213	0.857	0.797	0.927	0.570	0.303	0.802	0.716	0.913	0.679	0.448	0.883	0.976	0.806	0.694	0.479

**Table 12:** Comparison for 5 selected images from Histological dataset based on external clustering criteria.

Algorithm	ih2ycmuhwrgalo					pbphl1xujdvyx					ebvubdfxocisgny					0anzqyibfuc					4nkj5wqcqj				
	Dice	Precision	Recall	ARI	AMI	Dice	Precision	Recall	ARI	AMI	Dice	Precision	Recall	ARI	AMI	Dice	Precision	Recall	ARI	AMI	Dice	Precision	Recall	ARI	AMI

CutESC	0.889	0.973	0.818	0.785	0.490	0.937	0.909	0.968	0.697	0.421	0.948	0.959	0.938	0.700	0.400	0.973	0.965	0.981	0.769	0.529	0.947	0.932	0.964	0.667	0.433
HDBSCAN	0.870	0.877	0.863	0.725	0.562	0.876	0.959	0.805	0.582	0.359	0.953	0.943	0.963	0.692	0.453	0.973	0.962	0.985	0.765	0.510	0.899	0.937	0.864	0.509	0.292
AUTOCLUST	0.681	0.539	0.925	0.032	0.026	0.906	0.888	0.925	0.563	0.313	0.929	0.936	0.922	0.578	0.324	0.971	0.969	0.973	0.758	0.527	0.913	0.889	0.938	0.421	0.309
GDD	0.689	0.530	0.987	−0.004	0.004	0.834	0.961	0.736	0.501	0.279	0.921	0.961	0.884	0.598	0.368	0.863	0.972	0.776	0.383	0.259	0.703	0.942	0.561	0.222	0.151
DBSCAN	0.856	0.876	0.837	0.701	0.516	0.900	0.837	0.974	0.422	0.211	0.951	0.935	0.969	0.669	0.496	0.973	0.959	0.987	0.753	0.499	0.930	0.906	0.956	0.533	0.298
MeanShift	0.894	0.881	0.906	0.770	0.626	0.799	0.950	0.689	0.431	0.244	0.949	0.955	0.942	0.694	0.519	0.957	0.969	0.945	0.679	0.464	0.937	0.896	0.982	0.530	0.284
OPTICS	0.870	0.857	0.884	0.718	0.600	0.899	0.839	0.967	0.425	0.210	0.945	0.958	0.933	0.683	0.441	0.972	0.963	0.982	0.759	0.491	0.910	0.939	0.882	0.543	0.315

## 2 Experimental design, materials, and methods

### 2.1 The CutESC algorithm with optional configurations The CutESC (Cut-Edge for Spatial Clustering) algorithm with a graph-based approach is presented in [1]. This novel algorithm performs clustering automatically for outliers, complex shapes and irregular densities without requiring any prior information and parameters. Additionally, users can provide their own parameters to tune the clustering process by setting two parameters for specific applications. CutESC-P refers to the parametric version of our algorithm, see Algorithm 1.

Algorithm 1 Pseudocode of the CutESC-P Algorithm.

**Figure:**
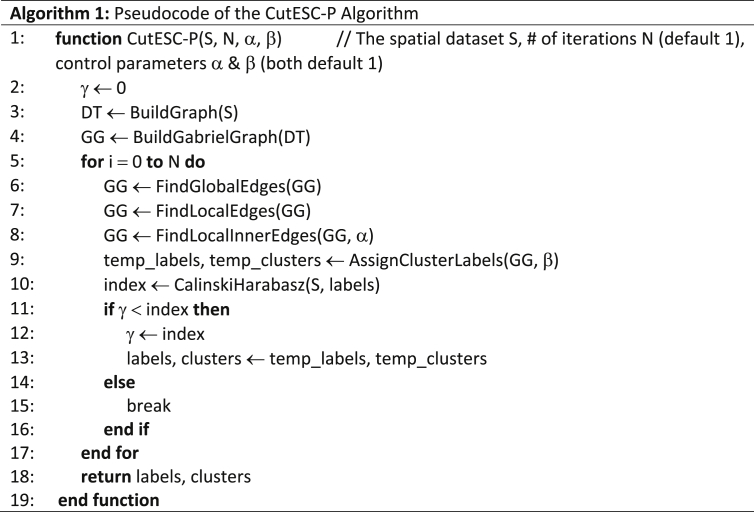

### 2.2 Various experiments on the CutESC algorithm

In this section, some additional information related to the CutESC algorithm is provided in detail. The presented algorithm includes 3-step thresholding procedures which should be applied in a hierarchy. In Fig. 1, the second and third thresholding rules of the CutESC algorithm are applied in a flipped order. Also, the CutESC algorithm can be computed iteratively. In Fig. 2, Fig. 3, the CutESC algorithm obtains the optimal solution in the first iteration (level 1). The relation between the levels/iterations is given in Table 1, where the number of clusters and Calinski- Harabasz score are shown for each level/iteration.

### 2.3 Selected parameters for several datasets

The best parameter settings for the experiments are given in this section. To find the best parameters, we scanned through combinations of values for each algorithm. In the pre-processing step, features are standardized by subtracting the mean and scaling to unit variance, and all features are centered around zero. The best parameters for 3-spiral [5], Aggregation [6], Compound [7], D31 [8], and Zelnik4 [9] datasets are given at Table 2. Table 3 shows the best parameters for Chameleon [3] dataset. Table 4 shows the best parameters for UCI (Dermatology, Ionosphere, Heart-Statlog, Cardiac-Arrhythmia, Thyroid-Allbp) [4] datasets. Table 5 shows the best parameters for BSDS500 [10] dataset. Finally, the best parameters for Histological [11] dataset are given at Table 6.

### 2.4 Additional experiments on external clustering criteria

External clustering criteria validate the experiments based on previous knowledge about data, when the ground truth data is known, and the predicted clusters are compared to the true one (see [1] for more details). Other details on external clustering criteria are reported in Table 7, Table 8. We can see that our method is highly competitive and outperforms other methods on some datasets in terms of external clustering criteria.

### 2.5 Additional experiments on multidimensional datasets

In this section, the additional analysis for Real-World datasets based on external clustering criteria is included. The comparison for Real-World datasets based on external clustering criteria is included in Table 9. Table 10 includes the number of instances that were attributed to each cluster as compared with the ground truth for Real-World datasets.

### 2.6 External clustering criteria for selected images from BSDS500 and histological datasets

In this section, the external clustering criteria of some selected images from these image segmentation datasets are given in Table 11, Table 12, where our algorithm outperforms other methods.
